# Antimicrobial Peptide TP4 Induces ROS-Mediated Necrosis by Triggering Mitochondrial Dysfunction in Wild-Type and Mutant *p53* Glioblastoma Cells

**DOI:** 10.3390/cancers11020171

**Published:** 2019-02-01

**Authors:** Bor-Chyuan Su, Chieh-Yu Pan, Jyh-Yih Chen

**Affiliations:** 1 Marine Research Station, Institute of Cellular and Organismic Biology, Academia Sinica, 23-10 Dahuen Road, Jiaushi, Ilan 262, Taiwan; su8265@gmail.com; 2 Department and Graduate Institute of Aquaculture, National Kaohsiung University of Science and Technology, Kaohsiung 811, Taiwan; panjade66@gmail.com

**Keywords:** antimicrobial peptide, tilapia piscidin 4, glioblastoma, *p53*, ROS, DNA damage, necrosis

## Abstract

Antimicrobial peptide tilapia piscidin 4 (TP4) from *Oreochromis niloticus* exhibits potent bactericidal and anti-tumorigenic effects. In a variety of cancers, the mutation status of p53 is a decisive factor for therapeutic sensitivity. Therefore, we investigated the impact of *p53* status on TP4-induced cytotoxicity in glioblastoma cell lines and the molecular mechanisms that govern cytotoxic effects. Both U87MG (wild-type/WT *p53*) and U251 (mutant *p53*) glioblastoma cell lines were sensitive to TP4-induced cytotoxicity. The necrosis inhibitors Necrostatin-1 and GSK’872 attenuated TP4-induced cytotoxicity, and TP4 treatment induced the release of cyclophilin A, a biomarker of necrosis. Moreover, TP4 induced mitochondrial hyperpolarization and dysfunction, which preceded the elevation of intracellular reactive oxygen species, DNA damage, and necrotic cell death in both U87MG and U251 glioblastoma cells. p38 was also activated by TP4, but did not contribute to cytotoxicity. SB202190, a specific p38 inhibitor, enhanced TP4-induced oxidative stress, mitochondrial dysfunction, and cytotoxicity, suggesting a protective role of p38. Furthermore, TP4-induced cytotoxicity, oxidative stress, phosphorylation of p38, and DNA damage were all attenuated by the mitochondrial-targeted reactive oxygen species (ROS) scavenger MitoTEMPO, or the reactive oxygen species scavenger N-acetyl-L-cysteine. Based on these data, we conclude that TP4 induces necrosis in both WT and mutant *p53* glioblastoma cells through a mitochondrial ROS-dependent pathway.

## 1. Introduction

Glioblastoma, a highly aggressive and invasive cancer, is the most common type of human brain cancer [1]. Current treatment for glioblastoma includes surgery, radiation therapy, and chemotherapy [2]. Unfortunately, even for patients that receive aggressive therapy, the five-year survival rate is less than 10% [3]. Mutations in *p53* are detected in 30–50% of glioblastoma cases and correlate with poor prognosis [4,5], with enhanced tumor growth, invasiveness, and metastasis [5]. In vitro studies have revealed that mutant *p53*-expressing glioblastoma cell lines are more sensitive to commonly used chemotherapeutic drugs, 1,3-bis(2-chloroethyl)-1-nitroso-urea (BCNU) and temozolomide (TMZ), than wild-type (WT) cells [6,7]. This observation may due to suppression of DNA repair protein O6-methylguanine methyltransferase (MGMT) in mutant *p53* glioblastoma cells [8] because MGMT expression is known to be regulated by functional *p53* [9]. Thus, the functional status of *p53* and its downstream targets is crucial for chemosensitivity in glioblastoma.

Antimicrobial peptide tilapia piscidin (TP)-4 was identified from Nile tilapia (*Oreochromis niloticus*) and exhibits strong bactericidal effects toward methicillin-resistant *Staphylococcus aureus*, *Streptococcus aglactiae*, *Vibrio vulnificus*, *Vibrio alginolyticus*, and *Helicobacter pylori* through disruption of the bacterial cell wall [10,11,12]. Interestingly, a recent study also demonstrated that TP4 displays anti-cancer function toward triple-negative breast cancer cells via FOSB targeting and induction of mitochondrial dysfunction [13]. However, the effect of TP4 on glioblastoma has not been previously studied. In the present study, we determined the effect of *p53* mutation status on TP4-induced cytotoxicity in glioblastoma cell lines. In addition, we investigated the underlying molecular mechanisms that contribute to TP4 cytotoxicity in both WT and mutant *p53* lines. We found that both WT and mutant *p53* glioblastoma cell lines are more sensitive to TP4 than non-cancerous cells. In glioblastoma cell lines, TP4 induces cell death via mitochondrial hyperpolarization and dysfunction, followed by increased reactive oxygen species production and resultant DNA damage and necrosis.

## 2. Results

### 2.1. TP4 Induces Death in Glioblastoma Cell Lines through a p53-Independent Mechanism

**Figure 1:**
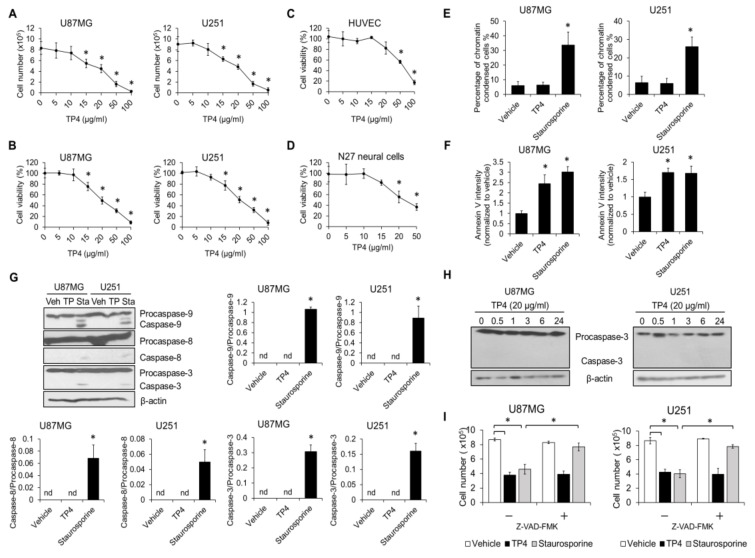
Caspase-mediated cell death is not induced by tilapia piscidin (TP) 4. U87MG (wild-type *p53*) and U251 (mutant *p53*) cells were treated with different doses of TP4 (0, 5, 10, 15, 20, 50, and 100 μg/mL) for 24 h. Cell number (**A**) and cell viability (**B**) were assessed. Cell number was determined using the trypan exclusion assay. Cell viability was assessed using the 3-(4,5-dimethylthiazol-2-yl)-5-(3-carboxymethoxyphenyl)-2-(4-sulfophenyl)-2H-tetrazolium inner salt)/phenazine methosulfate (MTS/PMS) assay. Human umbilical vein endothelial cells (HUVECs) (**C**) and N27 rat neural precursor cells (**D**) were treated with indicated doses of TP4 for 24 h. Cell viability was assessed using MTS/PMS assay. U87MG and U251 cells were treated with TP4 (20 μg/mL) and staurosporine (1 μM) for 24 h and 6 h, respectively. Apoptosis was monitored by chromatin condensation (**E**), Annexin V binding (**F**), and caspase activation (**G**). Condensed nuclei were scored from 4′,6-diamidino-2-phenylindole (DAPI)-stained cells. Intensity of Annexin V-Fluorescein isothiocyanate (FITC) was measured by flow cytometry. Cell lysates were collected and immunoblotted with caspase-3, -8 and -9 antibodies. β-actin was used as a loading control. Right and bottom panel: ratio of cleaved caspase-3, -8, and -9 to procaspase-3, -8, and -9, respectively. Veh: vehicle; TP: TP4; Sta: staurosporine. (**H**) Cells were treated with TP4 (20 μg/mL) for different times. Cell lysates were immunoblotted with caspase-3 antibody. (**I**) Cells were preincubated with pan-caspase inhibitor Z-VAD fluoromethylketone (Z-VAD-FMK) (100 μM) for 1 h, followed by TP4 (20 μg/mL) and staurosporine treatment for 24 h and 6 h, respectively. Cell number was determined using the trypan exclusion assay. Vehicle: 0.5% dimethyl sulfoxide (DMSO). Western blotting experiments were performed at least three times with similar results. Band intensities were quantified with Image J software (1.51j8; NIH, Bethesda, MD, USA). * *p* < 0.05, *n* = 3 for all groups. nd: not detectable.

p53 function is a critical mediator of chemosensitivity [14]. However, the effect of p53 mutation on antimicrobial peptide-induced cytotoxicity in cancer cells has not been previously reported. Here, we determined the role of *p53* in TP4-induced cytotoxicity to glioblastoma cell lines. Glioblastoma lines U87MG (WT *p53*) and U251 (mutant *p53*) were treated with various doses of TP4 (0–100 μg/mL), and cell viability and number were analyzed. The status of *p53* in U87MG and U251 cells was confirmed by probing Ser15 phosphorylation of p53 and accumulation of p53 and p21 after TP4 treatment. TP4 stabilized p53, induced Ser15 phosphorylation of p53, and caused p21 accumulation in U87MG (*p53* wild-type) cells but not in U251 (mutant *p53* cells) (Supplementary Figure S1). In addition, TP4 dose-dependently reduced cell viability and cell number in both U87MG and U251 cells (Figure 1A,B). The 50% lethal dose (LD50) of TP4 for both U87MG and U251 cells was 20 μg/mL. Most importantly, in both human umbilical vein endothelial cells (HUVECs) (Figure 1C) and N27 cells (Figure 1D), the LD50 for TP4 was found to be 50 μg/mL, suggesting that TP4 is more toxic to glioblastoma cells than normal cells.

### 2.2. TP4 Induces Caspase-Independent Cytotoxicity in Glioblastoma Cells

Since it has been shown that apoptosis is the major cell death pathway induced by chemotherapeutic agents [15], we evaluated parameters related to the induction of apoptosis in TP4-treated U87MG and U251 cells. Chromatin condensation, extracellular phosphatidylserine exposure, and caspase activation were all assessed. Results showed that administration of the apoptotic stimulator, staurosporine, caused an increase in the percentage of cells with chromatin condensation in either U87MG or U251 cultures but TP4 did not (Figure 1E). To further explore the mechanism of cell death, we labeled cells with annexin V-FITC and found that the signal was elevated by both TP4 and staurosporine treatments (Figure 1F). Next, we evaluated the activation of caspases, including caspase-3, -8, and -9. U87MG and U251 cells were incubated with 20 μg/mL TP4 for 24 h, and cell lysates were immunoblotted with caspase-3, -8, and -9 antibodies. Activation of caspase-3, -8, or -9 was induced by staurosporine but not TP4 (Figure 1G). We also assessed whether apoptosis may occur early after TP4 treatment. In order to do so, U87MG and U251 cells were incubated with TP4 for different times. Results clearly showed that caspase-3 is not activated upon TP4 stimulation (Figure 1H). Furthermore, the pan-caspase inhibitor, Z-VAD-FMK, rescued cells from staurosporine-induced cytotoxicity, but did not attenuate the TP4-induced reduction of cell number (Figure 1I). Together, these results suggest that caspase-dependent cell death may not be the major route of cell death induced by TP4 in glioblastoma cells, at least within 24 h of treatment. 

### 2.3. Autophagy Is not Activated by TP4 in Glioblastoma Cell Lines

**Figure 2:**
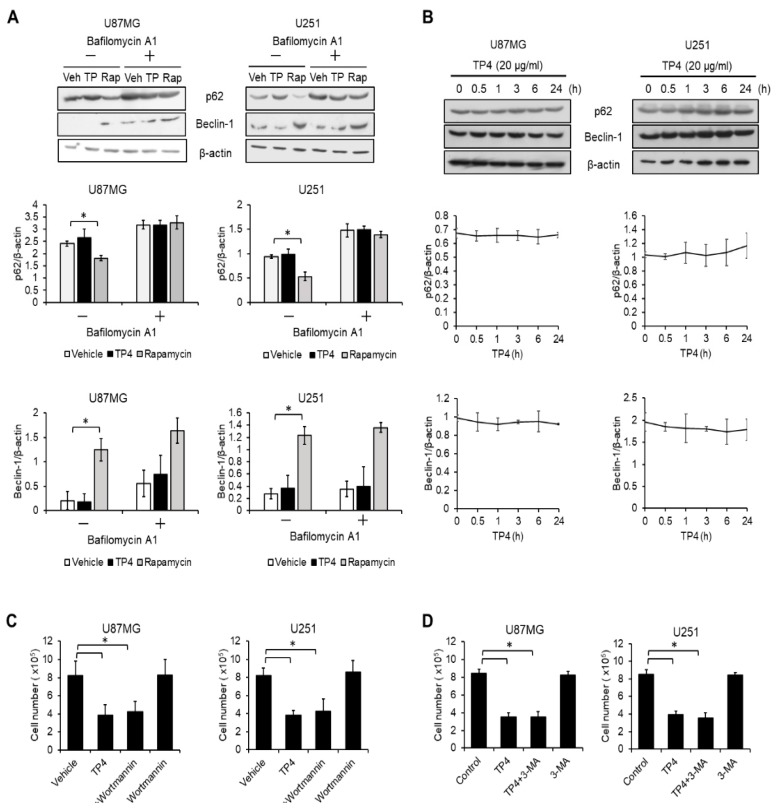
Autophagy is not affected by TP4. (**A**) Cells were treated with TP4 (20 μg/mL) and rapamycin (100 nM) with or without Bafilomycin A1 (100 nM) for 24 h. Cell lysates were immunoblotted with p62 and Beclin-1 antibodies. β-actin was used as a loading control. Veh: vehicle; TP: TP4; Rap: rapamycin. Bottom panel: quantification of p62 and Beclin-1. (**B**) Cells were treated with TP4 (20 μg/mL) for different times. Cell lysates were immunoblotted with p62 and Beclin-1 antibodies. β-actin was used as a loading control. Bottom panel: quantification of p62 and Beclin-1. Cells were preincubated with autophagy inhibitor, wortmannin (100 nM) (**C**) or 3-MA (5 mM) (**D**) for 1 h, followed by TP4 (20 μg/mL) treatment for 24 h. Cell number was determined using the trypan exclusion assay. Vehicle: 0.5% DMSO. Western blotting experiments were performed at least three times with similar results. Band intensities were quantified with Image J software. * *p* < 0.05, *n* = 3 for all groups. “–”: no treatment, “+”: TP4.

**Figure 3:**
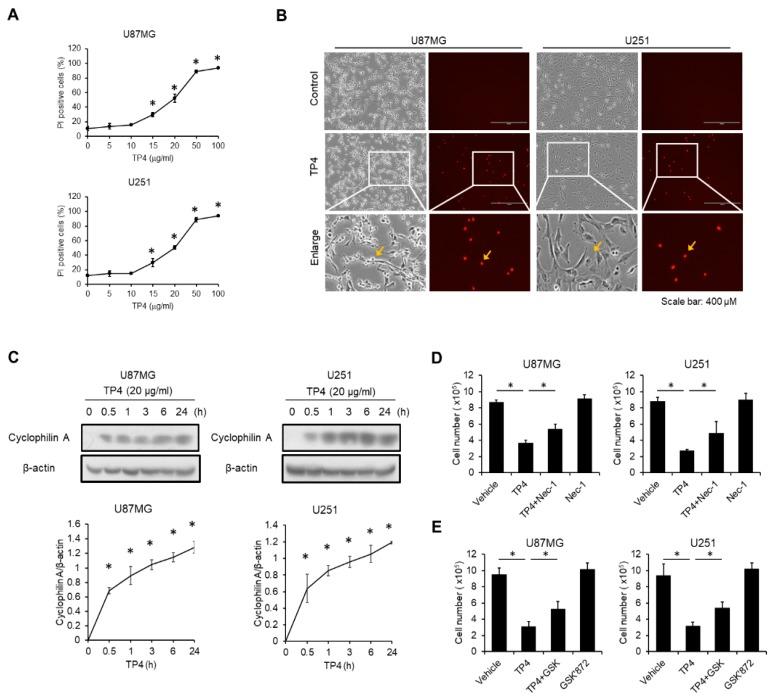
TP4 induces necrotic cell death in glioblastoma cells. Cells were treated with different doses of TP4 (0, 5, 10, 15, 20, 50 and 100 μg/mL) for 24 h. Necrosis was monitored by propidium iodide (PI) uptake. (**A**) The percentage of PI^+^ cells was assessed with flow cytometry. (**B**) PI uptake was observed, and images were captured with a fluorescence microscope. Cells were treated with TP4 (20 μg/mL) for 6 h. Yellow arrow indicates PI^+^ cells. Scale bar, 400 μm. (**C**) Cells were treated with TP4 (20 μg/mL) for different times. Supernatants were collected and immunoblotted with cyclophilin A antibody. Cell lysates were collected and immunoblotted with β-actin antibody. Bottom panel: quantification of cyclophilin A. Cells were preincubated with necrosis inhibitor Necrostatin-1 (Nec-1; 10 μM) (**D**) or GSK’872 (5 μM) (**E**) for 1 h, followed by TP4 (20 μg/mL) treatment for 24 h. Cell number was determined using the trypan exclusion assay. Vehicle: 0.5% DMSO. Western blotting experiments were performed at least three times with similar results. Band intensities were quantified with Image J software. * *p* < 0.05, *n* = 3 for all groups.

Since autophagy is considered to be another major programmed cell death pathway [16], we next assessed whether it participates in TP4-mediated cytotoxicity. U87MG and U251 cells were treated with TP4 or the autophagy inducer rapamycin. We found that p62 was reduced upon exposure to rapamycin. In addition, Beclin-1 was increased by rapamycin. In contrast, the levels of p62 and Beclin-1 were not affected by TP4 (Figure 2A). Moreover, to evaluate autophagic flux, cells were treated with the autophagosome/lysosome fusion inhibitor, bafilomycin A1, followed by TP4 or rapamycin. Bafilomycin A1 inhibited rapamycin-induced degradation of p62, however, the levels of p62 and Beclin-1 were not affected by the combination of TP4 and Bafilomycin A1. In order to assess whether autophagy may occur early after TP4 treatment, U87MG and U251 cells were treated with TP4 for different times. Results demonstrated that autophagy markers, including p62 and Beclin-1, were not affected after TP4 treatment (Figure 2B). Correspondingly, the autophagy inhibitors wortmannin (Figure 2C) and 3-methyladenine (Figure 2D) did not ameliorate the TP4-induced reduction of cell number. From these results, we suggest that autophagy is also probably not involved in TP4-induced cell death in our observation time window.

### 2.4. TP4 Induces Necrotic Cell Death in Glioblastoma Cell Lines

Since we had already excluded the involvement of caspase-dependent cell death and autophagy in TP4-mediated cytotoxicity, we further determined whether TP4-treated cells undergo necrosis. To assess necrosis, cells were loaded with the membrane-impermeable intercalating fluorescent dye, propidium iodide (PI), after exposure to different doses of TP4. We found that percentage of PI^+^ cells was increased by TP4 stimulation in a dose-dependent manner for both U87MG and U251 cells (Figure 3A,B). Cyclophilin A, an early stage marker of necrosis, is known to be released when the cell membrane becomes permeable [17]. We found that the level of cyclophilin A in the cell supernatant was increased after exposure to TP4 at multiple time points (0.5, 1, 3, 6 and 24 h) (Figure 3C). Furthermore, necrosis inhibitors Necrostatin-1 (Nec-1) (Figure 3D) and GSK’872 (Figure 3E) effectively decreased TP4-mediated cytotoxicity in both U87MG and U251 cells. These data suggest that TP4 induces necrosis in glioblastoma cells.

### 2.5. TP4 Elevates Intracellular Reactive Oxygen Species (ROS)

**Figure 4:**
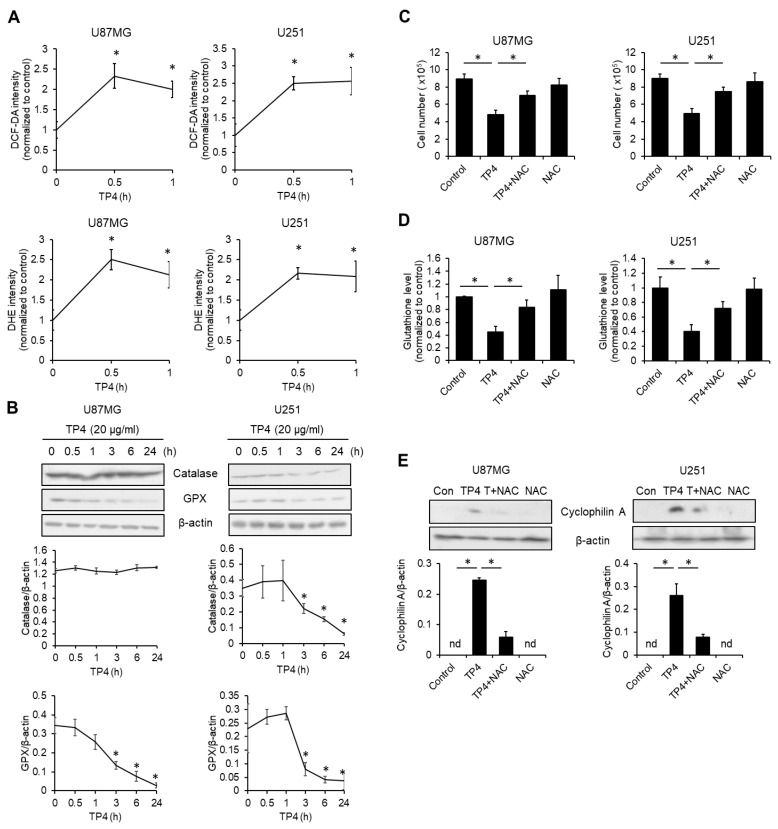
Reactive oxygen species (ROS) are required for TP4-induced necrotic cell death. (**A**) Cells were treated with TP4 (20 μg/mL) for 0, 0.5, and 1 h, followed by loading with 2’,7’-dichlorofluorescin diacetate (DCF-DA; 10 μM) or dihydroethidium (DHE; 20 μM) for 20 min. The fluorescence intensities of DCF-DA and DHE were analyzed using flow cytometry. (**B**) Cells were treated with TP4 (20 μg/mL) for different times. Cell lysates were collected and immunoblotted with the indicated antibodies. GPX: glutathione peroxidase. Bottom panel: quantification of catalase and GPX. (**C**) Cells were preincubated with ROS scavenger N-acetyl-L-cysteine (NAC; 5 mM) for 1 h, followed by TP4 (20 μg/mL) treatment for 24 h. Cell number was determined using the trypan exclusion assay. (**D**) Cells were preincubated with ROS scavenger NAC (5 mM) for 1 h, followed by TP4 (20 μg/mL) treatment for 3 h. The glutathione level was determined using the glutathione colorimetric assay kit. (**E**) Cells were treated as described for (**C**). Supernatants were immunoblotted with cyclophilin A antibody. Cell lysates were immunoblotted with β-actin antibody. Western blotting experiments were performed at least three times with similar results. Band intensities were quantified with Image J software. * *p* < 0.05, *n* = 3 for all groups. nd: not detectable.

Excessive free radical production is known to induce necrotic cell death [16], so we tested whether ROS is induced by TP4. Using ROS indicators 2’,7’-dichlorofluorescin diacetate (DCF-DA) and dihydroethidium (DHE), we found that TP4 treatment quickly led to elevated ROS levels. After treatment, the fluorescent signal was upregulated within 30 min and this elevation was sustained up to 1 h in both U87MG and U251 cells (Figure 4A). Administration of TP4 also suppressed the levels of antioxidant proteins (Figure 4B). In U87MG cells, glutathione peroxidase (GPX) was decreased at 3, 6, and 24 h after treatment with TP4, whereas catalase was not affected. In U251 cells, both catalase and GPX were diminished from 3 to 24 h after TP4 treatment. Next, we evaluated whether ROS elevation is necessary for TP4-mediated cytotoxicity. Cells were preincubated with the ROS scavenger, N-acetyl-L-cysteine (NAC), followed by treatment with a cytotoxic dose of TP4. NAC significantly inhibited TP4-induced reduction of cell number (Figure 4C), downregulation of glutathione levels (Figure 4D), and cyclophilin A release (Figure 4E). Together, these results suggest that ROS play an essential role in TP4-mediated cytotoxicity.

### 2.6. TP4 Impairs Mitochondria, Resulting in ROS Accumulation

**Figure 5:**
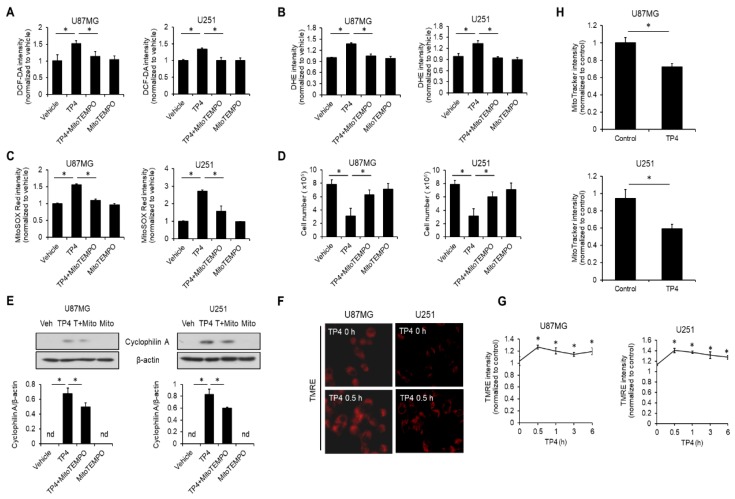
TP4 causes mitochondrial dysfunction and subsequently induces ROS production. Cells were preincubated with mitochondrial ROS scavenger MitoTEMPO (10 μM) for 1 h, followed by TP4 (20 μg/mL) for 1 h. ROS were labeled using DCF-DA (10 μM) (**A**) and DHE (20 μM) (**B**). The fluorescence intensities of DCF-DA and DHE were analyzed using flow cytometry. Vehicle: 0.5% DMSO. (**C**) Cells were preincubated with MitoTEMPO (10 μM) for 1 h, followed by TP4 (20 μg/mL) for 1 h. Afterward, cells were stained with a specific mitochondrial ROS indicator, MitoSOX Red, and fluorescence intensity was assessed using flow cytometry. Vehicle: 0.5% DMSO. (**D**) Cells were preincubated with MitoTEMPO (10 μM) for 1 h, followed by TP4 (20 μg/mL) treatment for 24 h. Cell number was determined using the trypan exclusion assay. Vehicle: 0.5% DMSO. (**E**) Cells were treated as described for (**D**). Supernatants were immunoblotted with cyclophilin A antibody. Cell lysates were immunoblotted with β-actin antibody. Veh: vehicle (0.5% DMSO); Mito: MitoTEMPO; T+Mito: TP4+MitoTEMPO. Bottom panel: quantification of cyclophilin A. (**F**) and (**G**) Cells were treated with TP4 (20 μg/mL) for indicated times, followed by loading with tetramethylrhodamine, ethyl ester (TMRE; 100 nM) for 15 min. The fluorescence intensity of TMRE was assessed using flow cytometry (**G**) and observed and images were captured with a fluorescence microscope (**F**). (**H**) Cells were treated with TP4 (20 μg/mL) for 0.5 h, followed by loading with MitoTracker Red CMXRos (100 nM) for 30 min. The fluorescence intensity of MitoTracker Red CMXRos was assessed using flow cytometry. Western blotting experiments were performed at least three times with similar results. Band intensities were quantified with Image J software. * *p* < 0.05, *n* = 3 for all groups. nd: not detectable.

Mitochondrial dysfunction often leads to excessive ROS production, which then may contribute to necrosis [18,19]. In order to test whether the source of TP4-induced ROS was mitochondria, cells were preincubated with a specific mitochondrial-ROS scavenger, MitoTEMPO, and then treated with TP4 for 0.5 h. After treatment, cells were probed with DCF-DA or DHE. Results showed that MitoTEMPO was able to ameliorate TP4-induced ROS accumulation (Figure 5A,B), suggesting that mitochondria are indeed the source of TP4-induced ROS. To confirm this finding, cells were treated with MitoTEMPO and TP4 as before, but the ROS level was specifically assessed in mitochondria by the indicator, MitoSOX Red. We found that TP4 treatment markedly elevated MitoSOX Red intensity, and this elevation could be attenuated by MitoTEMPO pretreatment (Figure 5C). In addition, MitoTEMPO attenuated TP4-induced cytotoxicity (Figure 5D) and cyclophilin A release (Figure 5E) in both U87MG and U251 cells. We then determined whether TP4 causes mitochondrial damage using the mitochondrial membrane potential indicator, tetramethylrhodamine, ethyl ester (TMRE), and MitoTracker Red CMXRos. As shown in Figure 5F,G, TMRE signals were quickly elevated after exposure to TP4 (within 30 min and sustained for 6 h), indicating that TP4 causes mitochondrial hyperpolarization. Moreover, administration of TP4 markedly induced a loss of MitoTracker Red CMXRos fluorescence in U87MG and U251 cells (Figure 5H), suggesting that mitochondrial function is impaired by TP4.

### 2.7. TP4 Induces a p38-Mediated Protective Effect

**Figure 6:**
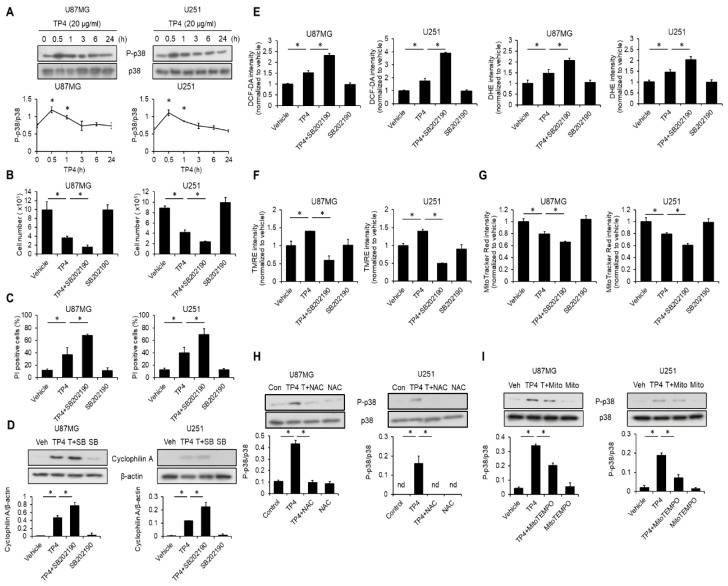
p38-mediated protective effect limits TP4-induced cytotoxicity. (**A**) Cells were treated with TP4 (20 μg/mL) for different times. Cell lysates were collected and immunoblotted with phospho-p38 (P-p38) and p38 antibodies. Bottom panel: quantification of P-p38. Cells were preincubated with a specific p38 inhibitor, SB202190 (20 μM) for 1 h, followed by TP4 (20 μg/mL) treatment for 24 h. Cell number was determined by the trypan exclusion assay (**B**), and the percentage of PI^+^ cells was assessed using flow cytometry (**C**) Vehicle: 0.5% DMSO. (**D**) Cells were treated as described for (**B**) and (**C**). Supernatants were immunoblotted with cyclophilin A antibody. Cell lysates were immunoblotted with β-actin antibody. Bottom panel: quantification of cyclophilin A. Veh (vehicle): 0.5% DMSO; SB: SB202190; T+SB: TP4+SB202190. (**E**) Cells were preincubated with SB202190 (20 μM) for 1 h, followed by TP4 (20 μg/mL) treatment for 1 h. ROS were labeled using DHE (20 μM) and DCF-DA (10 μM). Fluorescence intensity was assessed using flow cytometry. Cells were preincubated with a specific p38 inhibitor, SB202190 (20 μM) for 1 h, followed by TP4 (20 μg/mL) treatment for 1 h. The fluorescence intensity of TMRE (**F**) and MitoTracker Red CMXRos (**G**) was assessed using flow cytometry. Cells were preincubated with ROS scavenger NAC (5 mM) (**H**) or MitoTEMPO (10 μM) (**I**) for 1 h, followed by TP4 (20 μg/mL) treatment for 0.5 h. Cell lysates were collected and immunoblotted with P-p38 (phospho-p38) and p38 antibodies. Bottom panel: quantification of P-p38. Con: control; Veh: vehicle (0.5% DMSO); Mito: MitoTEMPO; T + Mito: TP4 + MitoTEMPO; T+NAC: TP4 + NAC. Western blotting experiments were performed at least three times with similar results. Band intensities were quantified with Image J software. * *p* < 0.05, *n* = 3 for all groups. nd: not detectable.

Mitogen activated protein kinase, p38, has been implicated in the regulation of necrosis [20]. As such, we tested whether p38 is activated and involved in TP4-mediated necrosis. U87MG and U251 cells were treated with TP4 for different times. We found that TP4 elevated p38 phosphorylation at 0.5 h after cells were exposed to TP4 (Figure 6A). Next, we assessed the involvement of p38 in TP4-induced necrosis. In this experiment, cells were preincubated with a specific p38 inhibitor, SB202190, followed by treatment with TP4. Results showed that SB202190 significantly enhanced TP4-induced loss of cell number (Figure 6B), increased the percentage of PI^+^ cells (Figure 6C), and elevated cyclophilin A release (Figure 6D). Thus, p38 appears to play a role in protecting against TP4-induced necrosis. Furthermore, cells that were preincubated with SB202190 followed by treatment with TP4 showed markedly enhanced TP4-induced ROS production (Figure 6E), and mitochondrial dysfunction (Figure 6F,G). TP4 alone, elevated TMRE intensity, while the combination of TP4 and SB202190 dramatically reduced TMRE intensity in U87MG and U251 cells (Figure 6F). In addition, SB202190 enhanced TP4-induced reduction of MitoTracker Red CMXRos fluorescence (Figure 6G), suggesting that p38 may act in a protective capacity to limit TP4-induced mitochondrial dysfunction and oxidative stress. Interestingly, when cells were preincubated with ROS scavenger, NAC, or mitochondrial-ROS scavenger, MitoTEMPO, followed by treatment with TP4, p38 phosphorylation was attenuated (Figure 6H,I), suggesting that TP4-elevated mitochondrial ROS is required for the induction of p38 phosphorylation (Figure 6I). Since phosphorylation of p38 limits TP4-induced mitochondrial dysfunction, ROS, and necrosis, the activation of p38 appears to be a compensatory defense mechanism.

### 2.8. TP4 Induces ROS-Mediated DNA Damage

**Figure 7:**
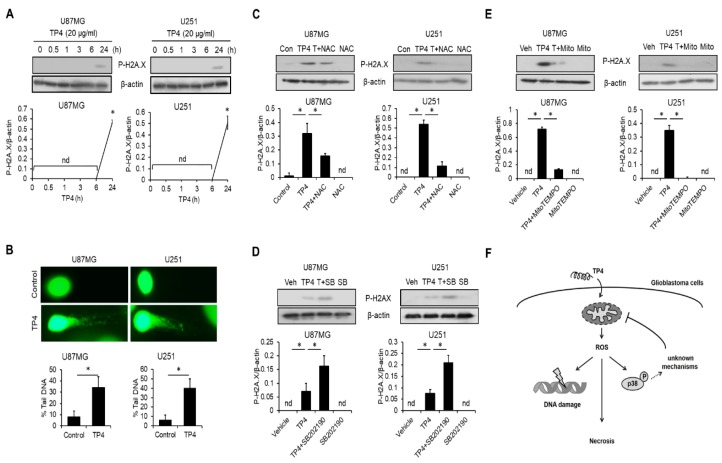
ROS are crucial for TP4-induced DNA damage. (**A**) Cells were treated with TP4 (20 μg/mL) for different times. Cell lysates were collected and immunoblotted with P-H2A.X (phospho-H2A.X) and β-actin antibodies. Bottom panel: quantification of P-H2A.X. (**B**) Comet assay results of TP4 exposure. Cells were treated with or without TP4 (20 μg/mL) for 24 h. Bottom panel: percentage of DNA tail was measured in 100 cells. Cells were preincubated with ROS scavenger NAC (5 mM), (**C**), MitoTEMPO (10 μM) (**D**) or SB202190 (20 μM) (**E**) for 1 h, followed by TP4 (20 μg/mL) treatment for 24 h. Cell lysates were collected and immunoblotted with P-H2A.X and β-actin antibodies. Bottom panel: quantification of P-H2A.X. Con: control; Veh: vehicle (0.5% DMSO); SB: SB202190; T + SB: TP4 + SB202190; Mito: MitoTEMPO; T + Mito: TP4 + MitoTEMPO; T + NAC: TP4 + NAC. Western blotting experiments were performed at least three times with similar results. Band intensities were quantified with Image J software. * *p* < 0.05, *n* = 3 for all groups. nd: not detectable. (**F**) Mechanisms underlying TP4-induced necrosis of glioblastoma cells. In wild-type (WT) and mutated *p53* glioblastoma cells, TP4 triggers mitochondrial hyperpolarization and dysfunction, subsequently inducing ROS production. Excessive ROS oxidize DNA to cause damage and also stimulate necrosis. In addition, ROS stimulates p38 phosphorylation, which limits TP4-induced cytotoxicity. p38 exerts a protective effect on mitochondria through unknown mechanisms.

Since DNA is a primary target of ROS [21], we also analyzed whether TP4 induces DNA damage in U87MG and U251 cells. To probe for a common marker of DNA damage-related signaling, cells were treated with TP4 for 0, 0.5, 1, 3, 6, or 24 h, and H2A.X immunoblotting was performed because H2A.X phosphorylation is a sensitive marker of DNA damage response [22]. Results demonstrated that H2A.X phosphorylation was increased after cells were exposed to TP4 for 24 h (Figure 7A). A comet assay further revealed that TP4 induces DNA strand breaks after cells were exposed to TP4 (Figure 7B). NAC (Figure 7C) and MitoTEMPO (Figure 7D) significantly reduced the accumulation of H2A.X phosphorylation, suggesting that ROS are essential for TP4-induced DNA damage. Furthermore, preincubation of cells with SB202190 strongly enhanced TP4-induced DNA damage (Figure 7E).

## 3. Discussion

Although many drugs have been identified and used to treat glioblastoma patients, their value in increasing progression-free survival or overall survival remains limited [2,3,23]. One reason for the limited efficacy of anti-glioblastoma drugs may be that a single glioblastoma lesion often contains both WT and mutated *p53* cancer cells [24]. Since the efficacy of conventional chemotherapeutic drugs in glioblastoma is largely dependent on the status of *p53* and its downstream targets, such as MGMT [4], this heterogeneity may cause drugs to be only partially effectual in many patients. WT *p53* glioblastoma cell lines tend to respond poorly to BCNU and TMZ compared to mutated *p53* glioblastoma lines [6,7], which increases the likelihood that resistance to chemotherapy will develop. Thus, there is an urgent need to develop new therapeutic drugs for glioblastoma that are effective against both WT and mutant *p53* cancer cells.

In this study, we found that TP4 differs from conventional chemotherapeutic drugs because it effectively induces necrosis in glioblastoma cells, independent of *p53* status. This feature may represent a major advantage in anti-glioblastoma therapy. However, glioblastoma is genetically a highly heterogeneous cancer, and several targets have been implicated in chemoresistance, including MGMT [25,26,27], isocitrate dehydrogenase (IDH) 1/2 [28], and epidermal growth factor receptor [29]. It is still unclear whether these factors influence TP4-mediated cytotoxicity in glioblastoma cells. Furthermore, a number of other factors are known to influence chemosensitivity of tumor cells, including the tumor microenvironment [30,31], invasion and stemness of cancer cells [31]. Moreover, hypoxia is a common feature of glioblastoma that is strongly correlated with radio- and chemoresistance through the enhancement of apoptosis-resistance and angiogenesis [32]. A hypoxic environment also promotes stem-cell reprogramming of non-stem cancer cells and facilitates self-renewal [33]. Glioblastoma stem cells contribute to the continuous growth of tumors, cancer recurrence and radio- and chemoresistance [33]. In addition, a hypoxic environment enhances tumor invasion by inducing epithelial-mesenchymal transition [34].

In general, conventional antimicrobial peptides stimulate apoptosis in cancer cells [35,36,37]. However, we found that TP4 induces necrosis in glioblastoma cells, and not caspase-dependent cell death or autophagy, at least within our observation time-window (24 h). This observation is consistent with a previous report that TP4 induces necrosis in triple negative breast cancer cells [13]. Interestingly, we found that TP4 increased the percentage of Annexin V-positive cells; however, caspases are not activated, and Z-VAD-FMK did not abolish TP4-induced cytotoxicity. TP4-induced elevation of Annexin V signal may be due to a disruption of cell membrane integrity, which would allow Annexin V penetrate the membrane and bind to phosphatidylserine on the inner leaflet. The use of cytotoxic agents to induce necrosis in cancer cells has been applied clinically as a therapeutic strategy [38]. High mobility group box 1 protein and cyclophilin A are both released from necrotic cancer cells, and may initiate antitumor immunity by enhancing migration and maturation of dendritic cells [17,39,40]. The mature dendritic cells can then facilitate the activation of natural killer cells and cytotoxic T lymphocytes, which may help to eliminate cancers [41,42]. It has also been demonstrated that targeting mitochondria can be a useful strategy for combatting cancers [43]. A recent study also demonstrated that a small-molecule inhibitor of transforming acidic coiled-coil containing protein 3, KHS101, effectively kills glioblastoma cells in vitro and in vivo by impairing mitochondrial metabolism and ATP production [44]. Additionally, targeting mitochondrial membrane protein voltage-dependent anion channels was shown to inhibit glioblastoma tumor growth and invasiveness through suppression of energy production and metabolic homeostasis [45]. Mechanistically, we found that TP4 quickly induces mitochondrial hyperpolarization, which results in mitochondrial dysfunction and increasing intracellular ROS production. Mitochondrial hyperpolarization usually leads to cell necrosis [46], while loss of mitochondrial polarization is linked to apoptosis [47,48]. Interestingly, we found that TP4 alone causes mitochondrial hyperpolarization, while the combination of TP4 and SB202190 results in mitochondrial hypopolarization and enhanced release of cyclophilin A. Moreover, mitochondrial dysfunction is often associated with ATP depletion and increased ROS generation [49]. Excessive ROS attacks cellular organelles and genetic material, leading to cell death [49]. We found that ROS induced by TP4 causes DNA damage and cell death in glioblastoma cells. 

Cancer cells often possess strong antioxidant defenses, which may help the cells to resist cytotoxic agents that induce oxidative stress [50]. Upon oxidative stress, cancer cells will initiate oncogenic K-Ras, B-Raf, and c-Myc, which are able to activate nuclear factor erythroid 2-related factor 2 (NRF2)-mediated anti-oxidant mechanisms [51]. Previous studies have shown that catalase and superoxide dismutase were greatly increased in TMZ-resistant glioblastoma cells [52]. We demonstrated that TP4 not only induces ROS production but also diminishes antioxidant defenses through the suppression of catalase and GPX, possibly rendering TP4 more cytotoxic in glioblastoma cell lines. The intracellular ATP level is an important factor that determines whether a cell will die by apoptosis or necrosis [53,54]. Preventing the depletion of intracellular ATP by 3-aminobenzamide (3-ABA) was previously shown to switch hydrogen peroxide-induced cell death from necrosis to apoptosis [54]. It is not clear whether TP4 depletes ATP in glioblastoma cells, and this leaves the interesting question of whether conservation of ATP levels by 3-ABA might modulate TP4-induced cell death. This and other future experiments will be conducted to further define the mechanisms that regulate cell death mediated by TP4. We also found that p38 MAPK is activated by mitochondrial ROS after TP4 treatment and that inhibition of p38 potentiates TP4-induced ROS, DNA damage, and necrosis in glioblastoma cells. These findings imply that activation of p38 functions to limit mitochondrial dysfunction and ROS generation, yet the exact mechanisms underlying this process remain unclear. A previous study showed that in mouse embryonic fibroblasts, p38 protects against oxidative stress-induced cell death by inducing the expression of antioxidant genes, including superoxide dismutase and catalase [55]. Upregulation of superoxide dismutase and catalase were not only required for eliminating oxidative stress, but also preserving mitochondrial function [56,57]. Regardless of the mechanism, inhibition of p38 by pharmacological or genetic approaches was previously shown to increase the sensitivity of glioblastoma cells to TMZ-induced cytotoxicity [58], suggesting that the p38 pathway plays a crucial role in the development of chemoresistance of glioblastoma cells.

Importantly, we also found that TP4 was more toxic to cancer cell lines than to non-cancerous HUVECs and N27 cells. This cytotoxicity preference may be a consequence of cancer cells displaying increased levels of phosphatidylserine on the cell surface compared to normal cells [59]. Increased phosphatidylserine exposure would be expected to attract the positively charged antimicrobial peptide, TP4, thereby providing a mechanism for TP4 to favor targeting to cancer cells. This feature of TP4-mediated cytotoxicity may be exploited to reduce the risk of undesirable side effects in potential TP4-derived chemotherapeutics. Although TP4 itself still causes cytotoxic effect in non-cancerous cells at higher doses, it is possible that the selectivity could be improved by introducing various chemical modifications [60]. The effects of chemically modified forms of TP4 will be tested in future studies.

Drugs used for glioblastoma therapy must be able to cross the blood–brain barrier, which leads to low therapeutic efficacy for many candidates [61]. However, several methods to circumvent this requirement have been recently developed. For example, the Ommaya reservoir has been used for glioblastoma treatment [62]. An Ommaya reservoir is an implanted device which allows chemotherapeutic drugs to be delivered into cerebrospinal fluid, where they can more easily reach cancer cells [63]. In another example, the Gliadel wafer, a biodegradable intracranial polymer implant, is able to provide slow release of BCNU to surrounding cells [64,65]. Although the ability of TP4 to cross the blood–brain barrier remains uninvestigated, its potential for treating glioblastoma is promising. If TP4 cannot penetrate the blood–brain barrier, it may either be injected into cerebral spinal fluid, using an Ommaya reservoir, or be administered as an intracranial polymer implant, similar to the Gliadel wafer.

## 4. Materials and Methods

### 4.1. Reagents

Tilapia piscidin 4 (TP4) peptide was synthesized by GL Biochem with the sequence, H-FIHHIIGGLFSAGKAIHRLIRRRRR-OH (Shanghai, China). 3-(4,5-dimethylthiazol-2-yl)-5-(3-carboxymethoxyphenyl)-2-(4-sulfophenyl)-2H-tetrazolium inner salt (MTS) and phenazine methosulfate (PMS) were purchased from Promega (Madison, WI, USA). Trypan blue, tetramethylrhodamine, ethyl ester (TMRE), MitoSOX™ Red (M36008), and MitoTracker™ Red CMXRos (M7512) were purchased from ThermoFisher (Waltham, MA, USA). Propidium iodide (PI), 2’,7’-dichlorofluorescin diacetate (DCF-DA), dihydroethidium (DHE), 4’,6-diamidino-2-phenylindole (DAPI), wortmannin, N-acetyl-L-cysteine (NAC), SB202190, Necrostatin-1 (Nec-1), staurosporine, 3-methyladenine (3-MA), bafilomycin A1, rapamycin, and MitoTEMPO were purchased from Sigma (Merck KGaA, Darmstadt, Germany). GSK’872 was purchased from BioVision (Milpitas, CA, USA). Z-VAD-FMK was purchased from Cell signaling Technology (Danvers, MA, USA).

### 4.2. Cell Culture

U87MG (WT *p53*) cells and human umbilical vein endothelial cells (HUVECs) were purchased from the Bioresource Collection and Research Center (Hsinchu, Taiwan). The N27 rat dopaminergic neural cell line was purchased from Millipore (Merck KGaA, Darmstadt, Germany). The U251 (mutant *p53*) cell line was a kindly provided by Dr. Pei-Jung Lu, Graduate Institute of Clinical Medicine, National Cheng Kung University, Tainan, Taiwan. U87MG and U251 cells were maintained in Dulbecco’s Modified Eagle’s medium (DMEM; Gibco, ThermoFisher, Waltham, MA, USA), supplemented with 10% fetal bovine serum (FBS; Gibco) and Antibiotic-Antimycotic (Gibco). HUVEC were maintained in ham’s F-12 (Gibco), supplemented with 10% FBS, heparin (Sigma) and endothelial cell growth factor (Sigma). The N27 cell line was maintained in Roswell Park Memorial Institute 1640 medium (RPMI; Gibco), supplemented with 10% FBS and antibiotic-antimycotic as previously described [66]. For TP4 treatment, TP4 was dissolved in distilled water and further diluted into medium. For the MTS/PMS assay, cells were seeded into 96-well plates at the density of 1.5 × 10^4^ per well. For Western blotting, cytotoxicity, ROS, and mitochondria functional assays, cells were seeded into 6-well plates at the density of 6 × 10^5^ per well.

### 4.3. Western Blotting

In order to assesses the release of cyclophilin A, culture supernatant was collected after treatment and was centrifuged to remove cell debris or suspended cells. Following centrifugation, 6X sample buffer was added to culture supernatant. To evaluate intracellular signaling, caspase activation, and autophagy markers, only attached cells (without culture supernatant) were collected and lysed by RIPA buffer (Merck). Cell lysates and supernatants were separated by SDS-page, and transferred onto PVDF membrane (GE Healthcare Life Sciences, Marlborough, MA, USA) for immunoblotting with indicated antibodies. Antibodies used in this study were purchased from Cell Signaling Technology: caspase-3 (9662), caspase-8 (4790), caspase-9 (9502), p62 (5114), eclin-1 (3738), cyclophilin A (2175), catalase (D5N7V), glutathione peroxidase (3206), phospho-p38 (9211), p38 (8690), phospho-H2A.X (9718), and β-actin (3700). In order to evaluate apoptosis, caspase-3, -8, and -9 antibodies that recognize both the procaspase and cleaved forms were used.

### 4.4. Annexin V-FITC Binding Assay

Cells were collected by centrifugation and then stained with annexin-V-FITC (eBioscience, ThermoFisher, Waltham, MA, USA) for 15 min at room temperature. Stained cells were analyzed by flow cytometry (Beckman Coulter, Indianapolis, IN, USA).

### 4.5. Cytotoxicity

Cell number was calculated by the trypan blue exclusion assay as previously described [67]. Propidium iodide (PI) uptake assay was performed as previously described with minor modifications [68]. Briefly, cells were stained with PI (1 μg/mL) for 10 min. Excess PI was washed out by phosphate buffer saline (PBS). After staining, cells were imaged by fluorescence microscopy (EVOS FL Cell Imaging System, ThermoFisher, Waltham, MA, USA). The percentage of cells with PI uptake was quantified by flow cytometry (Beckman Coulter). Cell viability was determined by the MTS/PMS assay, according to the manufacturer’s instructions. Briefly, cells were loaded with the MTS/PMS mixture and incubated in the plate for 1 h at 37 °C. Absorbance at 490 nm was measured using a microplate reader. In order to evaluate chromatin condensation, cells were fixed in 10% formalin and stained with DAPI. Condensed nuclei in eight random fields were scored under fluorescence microscopy.

### 4.6. ROS and Mitochondrial Functional Analysis

Cells were loaded with the ROS indicators, DHE (20 μM) or DCF-DA (10 μM), for 20 min. After loading, cells were rinsed with PBS. Fluorescence intensities of DCF-DA (excitation/emission: 490/520 nm) and DHE (excitation/emission: 530/575 nm) were analyzed by flow cytometry (Beckman Coulter). Mitochondrial ROS was evaluated with MitoSOX Red. Following treatment, cells were loaded with MitoSOX Red for 20 min. After loading, cells were rinsed with PBS. Fluorescence intensity of MitoSOX Red (excitation/emission: 530/575 nm) was analyzed by flow cytometry (Beckman Coulter). In order to assess mitochondrial membrane potential and mitochondrial function, TP4-treated cells were stained with TMRE (100 nM; excitation/emission: 530/575 nm) for 15 min or MitoTracker Red CMXRos (100 nM; excitation/emission: 530/575 nm) for 30 min, respectively. Only functional mitochondria could be labeled by MitoTracker Red CMXRos [69]. Fluorescence intensities of TMRE or MitoTracker Red were detected using flow cytometry. The intracellular levels of glutathione were assessed using a glutathione colorimetric assay kit (BioVision, K261), following the manufacturer’s instructions.

### 4.7. Comet Assay

DNA damage was measured by the Comet assay kit (Cell Biolabs, STA-355, San Diego, CA, USA), according to the manufacturer’s instructions. The percentage of tail DNA was quantified according to a previous study [70].

### 4.8. Statistical Analysis

All assays were performed in triplicate with at least three independent replicates. GraphPad Prism 5.0 software (San Diego, CA, USA) was used for statistical analysis. Data are shown as mean ± SEM. Data were analyzed by one-way ANOVA or Student’s *t*-test, as appropriate. *p* < 0.05 was considered statistically significant. *p*-values of each comparison are shown in the Table S1.

## 5. Conclusions

Overall, our study suggests that TP4 kills glioblastoma cell lines independent of *p53* mutation status by inducing mitochondrial hyperpolarization and mitochondrial dysfunction, followed by increased ROS production, which then causes DNA damage and necrosis in glioblastoma cell lines (summarized in Figure 7F). Based on our findings, further experiments should be undertaken using a larger variety of cell models and cell culture conditions (e.g., hypoxia), extended investigation timelines, and additional functional assays to measure mitochondrial dysfunction. In addition, the effects of TP4 should be evaluated in patient-derived glioblastoma cells to test the relevance of our findings to clinical disease. Experiments such as these would strengthen the notion that TP4 affects mitochondrial biology in glioblastoma. Nevertheless, TP4 may be considered as an intriguing lead compound for the development of glioblastoma treatments.

## Supplementary Materials

The following are available online at http://www.mdpi.com/2072-6694/11/2/171/s1, Figure S1: Accumulation and activation of p53 and p21 was only induced in U87MG cells not U251 after exposure to TP4, Table S1: *p*-values for each comparison.
