# Supplementary material for: Antimicrobial Peptide TP4 Induces ROS-Mediated Necrosis by Triggering Mitochondrial Dysfunction in Wild-Type and Mutant p53 Glioblastoma Cells
Source: Cancers (Basel). 2019 Feb 1;11(2):171. doi: 10.3390/cancers11020171 (PMC6406555; doi:10.3390/cancers11020171)
Supplement: Supplementary file 1 [file cancers-11-00171-s001.pdf]

Supplementary Materials: Antimicrobial Peptide TP4 Induces ROS-Mediated Necrosis by Triggering Mitochondrial Dysfunction in Wild-Type and Mutant *p53* Glioblastoma Cells

Bor-Chyuan Su, Chieh-Yu Pan and Jyh-Yih Chen

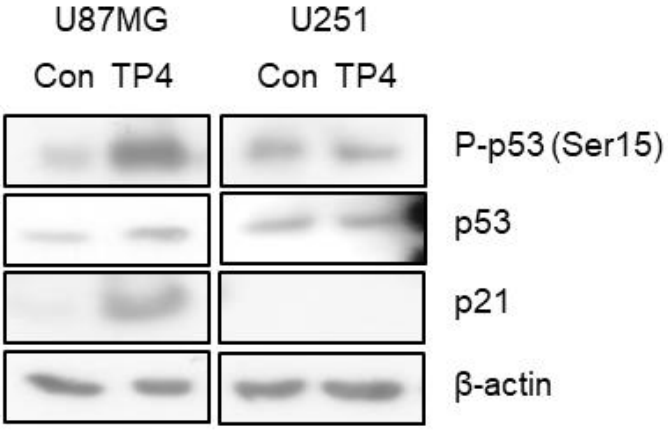

Figure S1. Accumulation and activation of p53 and p21 was only induced in U87MG cells not U251 after exposure to TP4. Cells were treated with TP4 (20 μg/mL) for 16 h, and cell lysates were immunoblotted with indicated antibodies.

Table S1. *p*-values for each comparison.

**Figure 1**			
Figure 1A-U87MG	*p*-value	Figure 1A-U251	*p*-value
0 vs. 5	0.9426	0 vs. 5	0.7297
0 vs. 10	0.4791	0 vs. 10	0.9964
0 vs. 15	0.0001	0 vs. 15	0.0001
0 vs. 20	0.0001	0 vs. 20	0.0001
0 vs. 50	0.0001	0 vs. 50	0.0001
0 vs. 100	0.0001	0 vs. 100	0.0001
Figure 1B-U87MG	*p*-value	Figure 1B-U251	*p*-value
0 vs. 5	0.6133	0 vs. 5	0.7556
0 vs. 10	0.9867	0 vs. 10	0.9965
0 vs. 15	0.0399	0 vs. 15	0.0001
0 vs. 20	0.0001	0 vs. 20	0.0001
0 vs. 50	0.0001	0 vs. 50	0.0001
0 vs. 100	0.0001	0 vs. 100	0.0001
Figure 1C-HUVEC	*p*-value	Figure 1D-N27	*p*-value
0 vs. 5	0.9953	0 vs. 5	0.9999
0 vs. 10	0.9983	0 vs. 10	0.9999
0 vs. 15	0.9046	0 vs. 15	0.5377
0 vs. 20	0.1740	0 vs. 20	0.0383
0 vs. 50	0.0001	0 vs. 50	0.0008
0 vs. 100	0.0001		
Figure 1E-U87MG	*p*-value	Figure 1E-U251	*p*-value
Veh vs. TP4	0.9699	Veh vs. TP4	0.6515

Veh vs. Sta	0.0004	Veh vs. Sta	0.0007
Figure 1F-U87MG	*p*-value	Figure 1F-U251	*p*-value
Veh vs. TP4	0.0014	Veh vs. TP4	0.0152
Veh vs. Sta	0.0002	Veh vs. Sta	0.0382
Figure 1G-U87MG Cas-9	*p*-value	Figure 1G-U251 Cas-9	*p*-value
Veh vs. TP4	0.9999	Veh vs. TP4	0.9999
Veh vs. Sta	0.0001	Veh vs. Sta	0.0001
Figure 1G-U87MG Cas-8	*p*-value	Figure 1G-U251 Cas-8	*p*-value
Veh vs. TP4	0.9999	Veh vs. TP4	0.9999
Veh vs. Sta	0.0001	Veh vs. Sta	0.0001
Figure 1G-U87MG Cas-3	*p*-value	Figure 1G-U251 Cas-3	*p*-value
Veh vs. TP4	0.9999	Veh vs. TP4	0.9999
Veh vs. Sta	0.0001	Veh vs. Sta	0.0001
Figure 1I-U87MG	*p*-value	Figure 1I-U251	*p*-value
Veh vs. TP4	<0.0001	Veh vs. TP4	<0.0001
Veh vs. Sta	<0.0001	Veh vs. Sta	<0.0001
Sta vs. Sta + Z-VAD	0.0001	Sta vs. Sta + Z-VAD	0.0001
**Figure 2**			
Figure 2A-U87MG p62	*p*-value	Figure 2A-U251 p62	*p*-value
Veh vs. TP4	0.0523	Veh vs. TP4	0.5330
Veh vs. Rap	0.0059	Veh vs. Rap	0.0492
Figure 2A-U87MG Beclin	*p*-value	Figure 2A-U251 Beclin	*p*-value
Veh vs. TP4	0.7766	Veh vs. TP4	0.9448
Veh vs. Rap	0.0014	Veh vs. Rap	0.0007
Figure 2C-U87MG	*p*-value	Figure 2C-U251	*p*-value
Veh vs. TP4	0.0274	Veh vs. TP4	0.0027
TP4 vs. TP4+Wort	0.9989	TP4 vs. TP4+Wort	0.8285
Veh vs. Wort	>0.9999	Veh vs. Wort	>0.9999
Figure 2D-U87MG	*p*-value	Figure 2D-U251	*p*-value
Con vs. TP4	<0.0001	Con vs. TP4	<0.0001
TP4 vs. TP4+3-MA	0.9989	TP4 vs. TP4+3-MA	0.8289
Con vs. 3-MA	>0.9999	Con vs. 3-MA	>0.9999
**Figure 3**			
Figure 3A-U87MG	*p*-value	Figure 3A-U251	*p*-value
0 vs. 5	0.7425	0 vs. 5	0.1019
0 vs. 10	0.2012	0 vs. 10	0.0542
0 vs. 15	0.0003	0 vs. 15	0.0001
0 vs. 20	0.0001	0 vs. 20	0.0001
0 vs. 50	0.0001	0 vs. 50	0.0001
0 vs. 100	0.0001	0 vs. 100	0.0001
Figure 3C-U87MG	*p*-value	Figure 3C-U251	*p*-value
0 vs. 0.5	0.0001	0 vs. 0.5	0.0001
0 vs. 1	0.0001	0 vs. 1	0.0001
0 vs. 3	0.0001	0 vs. 3	0.0001
0 vs. 6	0.0001	0 vs. 6	0.0001
0 vs. 24	0.0001	0 vs. 24	<0.0001
Figure 3D-U87MG	*p*-value	Figure 3D-U251	*p*-value
Veh vs. TP4	<0.0001	Veh vs. TP4	<0.0001
TP4 vs. TP4 + Nec-1	0.0001	TP4 vs. TP4 + Nec-1	0.0099
Veh vs. Nec-1	0.5535	Veh vs. Nec-1	0.7903

Figure 3E-U87MG	*p*-value	Figure 3E-U251	*p*-value
Veh vs. TP4	<0.0001	Veh vs. TP4	<0.0001
TP4 vs. TP4 + GSK	0.0310	TP4 vs. TP4 + GSK	0.0258
Veh vs. GSK	0.9983	Veh vs. GSK	0.9988
**Figure 4**			
Figure 4A-U87MG-DCF	*p*-value	Figure 4A-U251-DCF	*p*-value
0 vs. 0.5	0.0016	0 vs. 0.5	0.0011
0 vs. 1	0.0114	0 vs. 1	0.0005
Figure 4A-U87MG-DHE	*p*-value	Figure 4A-U251-DHE	*p*-value
0 vs. 0.5	0.0013	0 vs. 0.5	0.0021
0 vs. 1	0.0245	0 vs. 1	0.0008
Figure 4B-U87MG-Catalase	*p*-value	Figure 4B-U87MG-GPX	*p*-value
0 vs. 0.5	0.9997	0 vs. 0.5	0.6484
0 vs. 1	0.9999	0 vs. 1	0.5399
0 vs. 3	0.9997	0 vs. 3	0.0004
0 vs. 6	0.9997	0 vs. 6	0.0001
0 vs. 24	0.9996	0 vs. 24	0.0001
Figure 4B-U251-Catalase	*p*-value	Figure 4B-U251-GPX	*p*-value
0 vs. 0.5	0.9251	0 vs. 0.5	0.6745
0 vs. 1	0.9967	0 vs. 1	0.4337
0 vs. 3	0.0480	0 vs. 3	0.0027
0 vs. 6	0.0123	0 vs. 6	0.0010
0 vs. 24	0.0006	0 vs. 24	0.0008
Figure 4C-U87MG	*p*-value	Figure 4C-U251	*p*-value
Con vs. TP4	<0.0001	Con vs. TP4	<0.0001
TP4 vs. TP4 + NAC	0.0027	TP4 vs. TP4 + NAC	<0.0001
Con vs. NAC	0.5640	Con vs. NAC	0.3102
Figure 4D-U87MG	*p*-value	Figure 4D-U251	*p*-value
Con vs. TP4	<0.0001	Con vs. TP4	<0.0001
TP4 vs. TP4 + NAC	0.0015	TP4 vs. TP4 + NAC	0.0024
Con vs. NAC	0.6783	Con vs. NAC	0.6181
Figure 4E-U87MG	*p*-value	Figure 4E-U251	*p*-value
Con vs. TP4	<0.0001	Con vs. TP4	<0.0001
TP4 vs. TP4 + NAC	<0.0001	TP4 vs. TP4 + NAC	<0.0001
Con vs. NAC	>0.9999	Con vs. NAC	>0.9999
**Figure 5**			
Figure 5A-U87MG DCF	*p*-value	Figure 5A-U251 DCF	*p*-value
Veh vs. TP4	0.0287	Veh vs. TP4	0.0192
TP4 vs. TP4+Mito	0.0137	TP4 vs. TP4 + Mito	0.0336
Veh vs. Mito	0.8159	Veh vs. Mito	>0.9999
Figure 5B-U87MG DHE	*p*-value	Figure 5B-U251 DHE	*p*-value
Veh vs. TP4	<0.0001	Veh vs. TP4	0.0006
TP4 vs. TP4 + Mito	<0.0001	TP4 vs. TP4 + Mito	0.0003
Veh vs. Mito	0.7666	Veh vs. Mito	0.3782
Figure 5C-U87MG	*p*-value	Figure 5A-U251	*p*-value
Veh vs. TP4	<0.0001	Veh vs. TP4	<0.0001
TP4 vs. TP4 + Mito	<0.0001	TP4 vs. TP4 + Mito	0.0030
Veh vs. Mito	0.4823	Veh vs. Mito	0.9910
Figure 5D-U87MG	*p*-value	Figure 5D-U251	*p*-value
Veh vs. TP4	<0.0001	Veh vs. TP4	<0.0001
TP4 vs. TP4 + Mito	<0.0001	TP4 vs. TP4 + Mito	<0.0001

Veh vs. Mito	0.5887	Veh vs. Mito	0.7148
Figure 5E-U87MG	*p*-value	Figure 5E-U251	*p*-value
Veh vs. TP4	<0.0001	Veh vs. TP4	<0.0001
TP4 vs. TP4 + Mito	<0.0001	TP4 vs. TP4+Mito	<0.0001
Veh vs. Mito	>0.9999	Veh vs. Mito	>0.9999
Figure 5G-U87MG	*p*-value	Figure 5G-U251	*p*-value
0 vs. 0.5	0.0001	0 vs. 0.5	<0.0001
0 vs. 1	0.0086	0 vs. 1	<0.0001
0 vs. 3	0.0211	0 vs. 3	<0.0001
0 vs. 6	0.0168	0 vs. 6	<0.0001
Figure 5H-U87MG	*p*-value	Figure 5H-U251	*p*-value
Con vs. TP4	0.0025	Con vs. TP4	0.0003
**Figure 6**			
Figure 6A-U87MG	*p*-value	Figure 6A-U251	*p*-value
0 vs. 0.5	<0.0001	0 vs. 0.5	<0.0001
0 vs. 1	<0.0001	0 vs. 1	<0.0001
0 vs. 3	0.9988	0 vs. 3	>0.9999
0 vs. 6	>0.9999	0 vs. 6	0.9748
0 vs. 24	0.9988	0 vs. 24	>0.9999
Figure 6B-U87MG	*p*-value	Figure 6B-U251	*p*-value
Veh vs. TP4	<0.0001	Veh vs. TP4	<0.0001
TP4 vs. TP4+SB	0.0023	TP4 vs. TP4+SB	0.0016
Veh vs. SB	0.7680	Veh vs. SB	0.4325
Figure 6C-U87MG	*p*-value	Figure 6C-U251	*p*-value
Veh vs. TP4	0.0032	Veh vs. TP4	0.0080
TP4 vs. TP4 + SB	<0.0001	TP4 vs. TP4 + SB	<0.0001
Veh vs. SB	>0.9999	Veh vs. SB	>0.9999
Figure 6D-U87MG	*p*-value	Figure 6D-U251	*p*-value
Veh vs. TP4	0.0003	Veh vs. TP4	<0.0001
TP4 vs. TP4 + SB	0.0012	TP4 vs. TP4 + SB	0.0005
Veh vs. SB	>0.9999	Veh vs. SB	0.9427
Figure 6E-U87MG DCF	*p*-value	Figure 6E-U251 DCF	*p*-value
Veh vs. TP4	0.0003	Veh vs. TP4	<0.0001
TP4 vs. TP4 + SB	<0.0001	TP4 vs. TP4 + SB	<0.0001
Veh vs. SB	0.9996	Veh vs. SB	0.9999
Figure 6E-U87MG DHE	*p*-value	Figure 6E-U251 DHE	*p*-value
Veh vs. TP4	<0.0001	Veh vs. TP4	<0.0001
TP4 vs. TP4 + SB	0.0001	TP4 vs. TP4 + SB	<0.0001
Veh vs. SB	0.9999	Veh vs. SB	0.9636
Figure 6F-U87MG	*p*-value	Figure 6F-U251	*p*-value
Veh vs. TP4	<0.0001	Veh vs. TP4	<0.0001
TP4 vs. TP4 + SB	<0.0001	TP4 vs. TP4+SB	<0.0001
Veh vs. SB	0.7122	Veh vs. SB	0.9793
Figure 6G-U87MG	*p*-value	Figure 6G-U251	*p*-value
Veh vs. TP4	0.0002	Veh vs. TP4	0.0002
TP4 vs. TP4 + SB	0.0004	TP4 vs. TP4+SB	0.0002
Veh vs. SB	>0.9999	Veh vs. SB	0.9963
Figure 6H-U87MG	*p*-value	Figure 6H-U251	*p*-value
Con vs. TP4	<0.0001	Con vs. TP4	<0.0001

TP4 vs. TP4 + NAC	<0.0001	TP4 vs. TP4 + NAC	<0.0001
Con vs. NAC	>0.9999	Con vs. NAC	>0.9999
Figure 6I-U87MG	*p*-value	Figure 6I-U251	*p*-value
Veh vs. TP4	<0.0001	Veh vs. TP4	<0.0001
TP4 vs. TP4 + Mito	<0.0001	TP4 vs. TP4 + Mito	<0.0001
Veh vs. Mito	0.9985	Veh vs. Mito	0.9942
**Figure 7**			
Figure 7A-U87MG	*p*-value	Figure 7A-U251	*p*-value
0 vs. 0.5	>0.9999	0 vs. 0.5	>0.9999
0 vs. 1	>0.9999	0 vs. 1	>0.9999
0 vs. 3	>0.9999	0 vs. 3	>0.9999
0 vs. 6	>0.9999	0 vs. 6	>0.9999
0 vs. 24	<0.0001	0 vs. 24	<0.0001
Figure 7B-U87MG	*p*-value	Figure 7B-U251	*p*-value
Con vs. TP4	0.014	Con vs. TP4	0.0045
Figure 7C-U87MG	*p*-value	Figure 7C-U251	*p*-value
Con vs. TP4	<0.0001	Con vs. TP4	<0.0001
TP4 vs. TP4 + NAC	0.0062	TP4 vs. TP4 + NAC	<0.0001
Con vs. NAC	>0.9999	Con vs. NAC	>0.9999
Figure 7D-U87MG	*p*-value	Figure 7D-U251	*p*-value
Veh vs. TP4	0.0372	Veh vs. TP4	0.0144
TP4 vs. TP4 + SB	<0.0001	TP4 vs. TP4+SB	<0.0001
Veh vs. SB	>0.9999	Veh vs. SB	>0.9999
Figure 7E-U87MG	*p*-value	Figure 7E-U251	*p*-value
Veh vs. TP4	<0.0001	Veh vs. TP4	<0.0001
TP4 vs. TP4 + Mito	<0.0001	TP4 vs. TP4 + Mito	<0.0001
Veh vs. Mito	>0.9999	Veh vs. Mito	>0.9999

© 2019 by the authors. Licensee MDPI, Basel, Switzerland. This article is an open access article distributed under the terms and conditions of the Creative Commons Attribution (CC BY) license (http://creativecommons.org/licenses/by/4.0/).
